# Androgen effect on connexin expression in the mammalian female reproductive system: A systematic review

**DOI:** 10.17305/bjbms.2019.4501

**Published:** 2020-08

**Authors:** Datu Agasi Mohd Kamal, Siti Fatimah Ibrahim, Mohd Helmy Mokhtar

**Affiliations:** Department of Physiology, Faculty of Medicine, Universiti Kebangsaan Malaysia (UKM), Kuala Lumpur, Malaysia

**Keywords:** Androgen, androgen blocker, connexin, ovary, female reproductive system

## Abstract

The functions of androgen and connexin in the mammalian female reproductive system are suggested to be related. Previous research has shown that androgen affects connexin expression in the female reproductive system, altering its function. However, no definitive conclusion on their cause-effect relationship has been drawn yet. In addition, a high prevalence of women with polycystic ovary syndrome (PCOS), who are characterized by elevated androgen levels and failure of ovulation, has prompted the studies on the relationship between androgen and connexin in the ovaries. This systematic review aims to investigate the effect of androgen on connexin expression in the mammalian female reproductive system. The literature search was conducted using the MEDLINE via EBSCOhost and the Scopus database and the following keywords: “androgen” or “testosterone” or “androgen blocker” or “anti-androgen” or “androstenedione” or “dehydroepiandrosterone” or “flutamide AND connexin” or “gap junction” or “cell junction”. We only considered in vitro and in vivo studies that involved treatment by androgen or androgen receptor blockers and measured connexin expression as one of the parameters. Our review showed that the exposure to androgen or androgen blocker affects connexin expression but not its localization in the mammalian ovary. However, it is not clear whether androgen downregulates or upregulates connexin expression.

## INTRODUCTION

### Androgen in the mammalian female reproductive system

Androgens are male sex hormones that are important for the function of the female reproductive system [1]. In women, androgens are produced in the adrenal glands and the ovaries, and they include dihydrotestosterone (DHT), testosterone, androstenedione, dehydroepiandrosterone, and dehydroepiandrosterone sulphate. Only DHT and testosterone bind to the androgen receptor (AR), while the others act as proandrogens and need to be converted to testosterone for their action. Testosterone is considered as the most potent androgen in women [2].

Fluctuations in plasma testosterone levels occur throughout the menstrual cycle and are related to changes in the expression of AR in the endometrium [1]. The effects of androgens are mediated through the AR, which belongs to the nuclear receptor subfamily 3 group C and primarily functions as DNA-binding transcription factor to regulate gene expression. The AR also utilizes the non-genomic pathway to exert the effects of androgen, through the modulation of cytoplasmic or cell membrane-bound regulatory proteins [3].

Androgens are involved in both normal and pathological states of the female reproductive system. Testosterone or DHT treatment of postpubertal gilts in the late follicular phase led to an increase in the ovulation rate [4]. The survival of human ovarian tissue *in vitro* was enhanced with DHT treatment [5]. On the other hand, DHT treatment of rats reduces the ovarian weight and oocyte yield [6]. A high concentration of androgens has been reported in women with polycystic ovary syndrome (PCOS), which is related to failure of ovulation [7]. This is because hyperandrogenism disrupts the communication between follicles and stroma, resulting in follicular arrest and disturbed ovulation [8]. Moreover, a high concentration of testosterone administered to rats has been reported to downregulate the endometrial receptivity markers in the endometrium of rats [9].

### Connexin expression in the mammalian female reproductive system

Connexins (Cx) are a group of homologous transmembrane proteins that form gap junctions in vertebrates. Intercellular gap junctions are channels that directly connect the cytoplasm of adjacent cells, which allows the exchange of small molecules and inorganic ions between the cells, mediating their electrical and metabolic coupling [10,11]. Various types of connexins are expressed in the mammalian female reproductive system, which plays an important role in its physiology. Cx43 and Cx37 are predominantly expressed in the ovarian follicles of humans and mice [12]. In both mice and humans, Cx43 forms gap junctions that connect granulosa cells [13,14]. In mice, Cx37 connects oocytes with the surrounding cumulus cells; while in humans, the location of Cx37 in the granulosa cells of fertile women is not clear [12]. In the endometrium in both rats and humans, Cx26 and Cx43 are the two major connexins [15-17]. In rats, Cx26 was found in the luminal epithelium and Cx43 in the stromal compartment [15]. Additionally, Cx26 and Cx43 were reported to be expressed in the myometrium in rats and humans [18,19].

Intercellular gap junctions between granulosa cells and oocytes facilitate the transfer of ions and molecules between the cells and thus have a crucial role in folliculogenesis and oogenesis [13,20]. For example, Cx37 and Cx43 are important for follicle development and oocyte growth in mammals [21]. In Cx37 or Cx43 deficient mice, folliculogenesis is arrested at the early stage, where oocytes fail to reach the meiotic stage [22,23]. Furthermore, Cx43 expression is increased during follicular development and decreased during follicular atresia [24]. Cx37 is a predominant connexin that makes up gap junctions in oocytes. These gap junctions connect granulosa cells to oocytes and are responsible for the direct transfer of nutrients, such as amino acids and glucose, as well as ions. Besides, Cx37 is important for the regulation of pH in oocytes as well as cyclic guanosine monophosphate that maintains oocytes in meiotic arrest [25,26]. Injection of carbenoxolone - a broad gap junction blocker to mice causes a delay in the implantation of blastocysts [27]. In addition, in cultured primary human endometrial stromal cells treatment with the gap junction blocker results in impaired decidualization [28].

### Androgen effect on connexin expression

The functions of androgen and connexin in the mammalian female reproductive system are suggested to be related. Previous research has shown that androgen affects connexin expression in the female reproductive system, altering its function. However, no definitive conclusion on their cause-effect relationship has been drawn yet. In addition, a high prevalence of women with PCOS, who are characterized by elevated androgen levels and failure of ovulation, has prompted the studies on the relationship between androgen and connexin in the ovaries. This systematic review aims to investigate the effect of androgen on connexin expression in the mammalian female reproductive system.

## MATERIALS AND METHODS

### Literature review

A systematic review of the literature was conducted to identify studies on the effects of androgen on connexin expression in the female reproductive system. The literature search was conducted using the MEDLINE via EBSCOhost (published between 1942 and October 2019) and the Scopus (published between 1941 and October 2019) database. The search was done with the following keywords: androgen* or testosterone* or androgen blocker or anti-androgen or androstenedione or dehydroepiandrosterone or flutamide AND connexin* or gap junction* or cell junction*.

### Study inclusion and exclusion criteria

The results were limited to studies that were published in the English language and only academic articles published from 2000 to 2019 were included. For this review, only studies that involved treatment by androgen or AR blockers were included with connexin expression as one of the parameters.

### Data extraction and management

The selection of papers involved two phases. In the first phase, the titles and abstracts were screened and any articles that did not match the inclusion criteria were excluded. In the second phase, the remaining papers were retrieved and screened thoroughly by three independent authors (S.F.I., H.M.M., and D.A.). Any differences in the opinion were resolved by the discussion between the authors.

The following data were recorded from the studies: the type and age of used samples, the treatment given to the subjects, the type of analyzed parameters and the method of analysis, and the results and conclusion of the studies.

## RESULTS

### Search results

**FIGURE 1:**
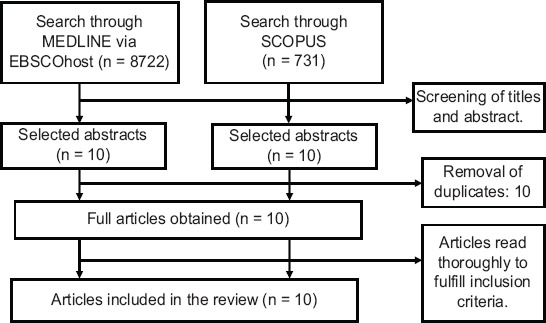
Flowchart of the literature selection process.

The literature search from two databases identified 20 articles. Ten articles were retrieved from MEDLINE and the other 10 from Scopus. Ten articles were removed due to duplication. For the remaining 10 articles, full texts were obtained for data extraction. The flowchart of the selection process is shown in Figure 1.

### Study characteristics

The characteristics of all studies are summarized in Table 1-3. Of the 10 eligible articles, three were *in vitro* studies that involved culture of oocytes derived from the Institute of Cancer Research (ICR) mice [29], Sprague Dawley rats [30], or humans [31]. Six articles were *in vivo* studies involving mice [24] or pigs [32-36]. One article combined both *in vitro* and *in vivo* studies on Sprague Dawley rats [37]. Nine studies were on ovaries or ovaries related tissue and cells, while one study involved the uterus and the placenta [36].

**TABLE 1:**
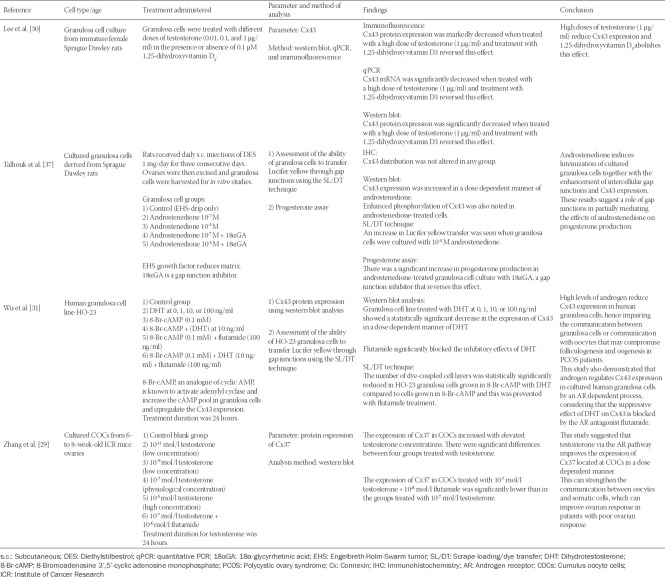
*In vitro* studies related to androgen effects on connexin expression in the mammalian female reproductive system

**TABLE 2:**
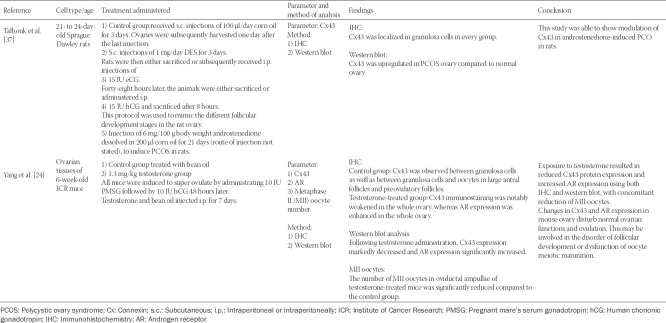
*In vivo* studies related to androgen effects on connexin expression in the mammalian female reproductive system

**TABLE 3:**
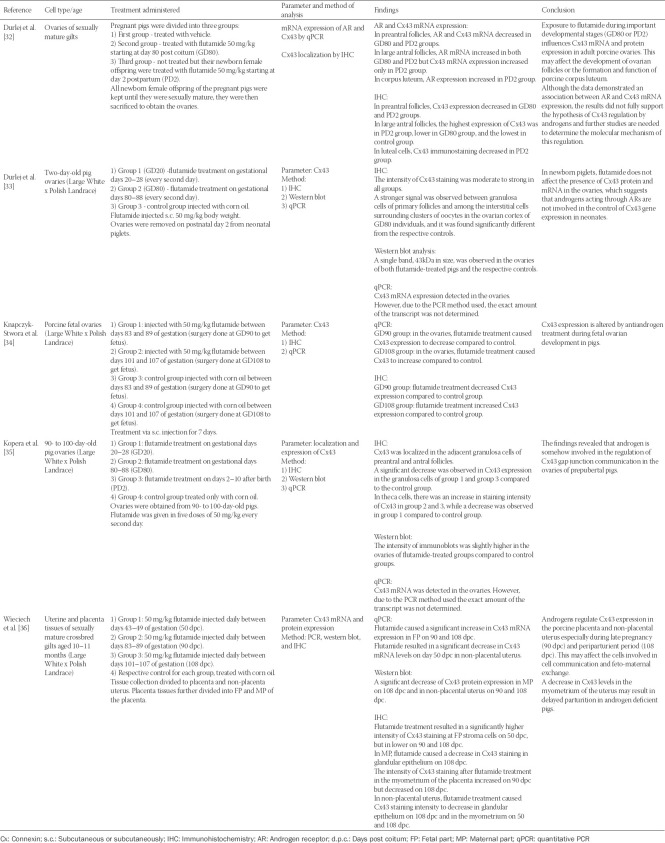
Studies related to AR blocker flutamide effects on connexin expression in the mammalian female reproductive system

All studies measured the expression of Cx43 as the parameter, except for Zhang et al. [29] who assessed Cx37 expression. Testosterone was used as treatment in three studies [24,29,30], one study used DHT [31], and one study used androstenedione [37]. The remaining five studies used the AR blocker flutamide as treatment [32-36].

Treatment by androgen decreased the expression of connexin in all studies except in the studies by Zhang et al. [29] and Talhouk et al. [37], which reported an increase in Cx37 after testosterone treatment and in Cx43 after androstenedione treatment, respectively. With flutamide treatment, connexins were reported to fluctuate according to the different gestation and postpartum period. The methods used to determine the expression of connexins included western blot, quantitative PCR, immunofluorescence, or immunohistochemistry. Two studies [31,37] used the scrape-loading/dye transfer (SL/DT) technique to assess the ability of granulosa cells to transfer Lucifer yellow through gap junctions.

## DISCUSSION

Based on our review, the results of studies investigating the effect of androgen or AR blocker treatment on connexin expression are inconsistent. About half of the included studies reported direct effects of androgens on connexin expression, while another half reported the effects of AR blockers on connexin expression. The studies involving AR blockers were in the prenatal period, postnatal period, or during gestation.

### *In vitro* studies

Four *in vitro* studies investigated androgen effects on connexin expression, with three studies using animal and one study using human cells. The three studies on animal cells showed contradictory results. Zhang et al. [29] showed increased expression of Cx37 in cultured cumulus-oocyte-cells (COCs) from ICR mice treated with testosterone. Similar findings were reported by Talhouk et al. [37], where Cx43 expression in the granulosa cells of rats was upregulated by androstenedione treatment. On the other hand, Lee et al. [30] showed that testosterone downregulated the expression of Cx43 in cultured granulosa cells derived from rats. In addition, the *in vitro* study using human granulosa cell lines revealed that Cx43 expression was decreased following DHT treatment and treatment with flutamide blocked this inhibitory effect on Cx43 [31].

Interestingly, Lee et al. [30] found that 1,25-dihydroxyvitamin D3 treatments of cultured granulosa cells reversed the inhibitory effects of testosterone on Cx43 expression. This finding suggests the use of 1,25-dihydroxyvitamin D3 as a treatment to prevent downregulation of Cx43. Wu et al. [31] and Talhouk et al. [37] used the SL/DT technique to assess the ability of granulosa cells to transfer Lucifer yellow through gap junctions. The Wu study showed that granulosa cells grown with DHT had a reduced ability to transfer Lucifer yellow dye, corresponding with Cx43 downregulation [31]. On the other hand, Talhouk et al. [37] showed an increase in Lucifer yellow transfer with the upregulation of Cx43 expression in androstenedione-treated cells. These studies confirmed the specific relationship between androgens and gap junctions composed of connexins. Furthermore, Talhouk et al. measured the production of progesterone by the cultured granulosa cells to determine the degree of cell differentiation. Their results showed that the progesterone production in androstenedione-treated cells increased parallel with the increase in Cx43 expression and Lucifer yellow transfer rate; whereas treatment with gap junction inhibitor 18α glycyrrhetinic acid (18αGA) reduced the progesterone yield. This suggests the role of gap junctions in mediating the effects of androstenedione on progesterone production [37].

With regards to androgen doses, all *in vitro* studies showed that the androgen effect on connexin expression is dose-dependent, except the study by Lee et al. [30], which showed that only high concentrations of testosterone (1 µg/ml) downregulated Cx43.

### *In vivo* studies

Only two *in vivo* studies involving direct androgen treatment were included in this review, showing contradictory results. Yang et al. [24] reported that testosterone reduced Cx43 expression in the ovaries of mice, while Talhouk et al. [37] showed that androstenedione increased Cx43 expression in the ovaries of rats. Moreover, in the Yang study, while Cx43 expression in the ovaries was reduced by testosterone treatment, the AR expression was upregulated [24]. This indicates that the effect of testosterone to downregulate the expression of Cx43 is mediated through the AR. Interestingly, this study also measured the number of metaphase II (MII) oocytes in the oviductal ampulla, showing a significantly reduced number of MII oocytes in testosterone-treated mice compared to control group. These results suggest that androgen affects connexin expression and results in follicular development disorder or dysfunction of oocyte meiotic maturation [24]. The Yang study considered 1.3 mg/kg testosterone as high-dose testosterone mimicking clinical feature of PCOS.

Talhouk et al. [37] induced PCOS by administrating androstenedione to rats and compared the level of connexin between the ovaries of normal rats and PCOS-induced rats. They showed that the expression of connexin was upregulated in the PCOS ovary compared to control. However, since androstenedione can be aromatized to estrogen, it is not clear whether the observed effect on connexin was induced by androstenedione or estrogen. Furthermore, androstenedione must be converted to testosterone or DHT to exert its effects [2].

### AR blocker

The five remaining studies [32-36] investigated the effects of flutamide, a potent AR blocker, on connexin expression. All five studies were *in vivo* studies using pigs as a model organism and the same concentration of flutamide (50 mg/kg). The studies considered prenatal, postnatal, neonatal, or during gestation exposure to flutamide. All studies were on the ovaries, except the study by Wieciech et al. [36], which involved the placenta and the uterus. These studies reported that the expression of Cx43 or AR varied in the ovaries, the placenta or the uterus depending on different stages (prenatal, postnatal, or neonatal), gestational days, or periods of exposure to flutamide. Four studies concluded that Cx43 expression is affected and regulated by anti-androgen flutamide treatment, while the study by Durlej et al. [33] indicated that Cx43 is not regulated by flutamide. None of the studies, however, explained the details on 50 mg/kg flutamide dose used.

This review shows that androgen or flutamide treatment does not affect the localization of Cx43. In addition, all included studies were focused on the effects of Cx37 or Cx43 downregulation on the ovary or uterus physiology, but none of the studies investigated the upregulation of Cx43 or Cx37 above the physiological expression levels, which may also disrupt the physiology of the female reproductive system.

## CONCLUSION

This review showed that the exposure to androgen or AR blockers affects the connexin expression but not its localization in the mammalian ovary. However, it is not clear whether androgen downregulates or upregulates connexin expression. In addition, most of the studies on the effects of androgen or AR blockers on connexin expression were conducted on the ovaries. Future studies should include other parts of the female reproductive system, especially the uterus, as women with PCOS that are characterized by high levels of androgens have shown defects in the uterus.
